# Sulfatide Preserves Insulin Crystals Not by Being Integrated in the Lattice but by Stabilizing Their Surface

**DOI:** 10.1155/2016/6179635

**Published:** 2016-02-14

**Authors:** Karsten Buschard, Austin W. Bracey, Daniel L. McElroy, Andrew T. Magis, Thomas Osterbye, Mark A. Atkinson, Kate M. Bailey, Amanda L. Posgai, David A. Ostrov

**Affiliations:** ^1^Bartholin Instituttet, Rigshospitalet, 2100 Copenhagen, Denmark; ^2^Department of Pathology, Immunology and Laboratory Medicine, University of Florida College of Medicine, 1600 SW Archer Road, Gainesville, FL 32610, USA

## Abstract

*Background*. Sulfatide is known to chaperone insulin crystallization within the pancreatic beta cell, but it is not known if this results from sulfatide being integrated inside the crystal structure or by binding the surface of the crystal. With this study, we aimed to characterize the molecular mechanisms underlying the integral role for sulfatide in stabilizing insulin crystals prior to exocytosis.* Methods*. We cocrystallized human insulin in the presence of sulfatide and solved the structure by molecular replacement.* Results*. The crystal structure of insulin crystallized in the presence of sulfatide does not reveal ordered occupancy representing sulfatide in the crystal lattice, suggesting that sulfatide does not permeate the crystal lattice but exerts its stabilizing effect by alternative interactions such as on the external surface of insulin crystals.* Conclusions*. Sulfatide is known to stabilize insulin crystals, and we demonstrate here that in beta cells sulfatide is likely coating insulin crystals. However, there is no evidence for sulfatide to be built into the crystal lattice.

## 1. Introduction

In beta cells of the islets of Langerhans, insulin is stored in granules as part of a crystalline insulin structure [1]. These molecular entities are formed within the Golgi apparatus known to contain zinc (Zn) molecules [1, 2]. Upon exocytosis into the bloodstream, the insulin crystals revert to a hexameric entity and subsequently transition into a monomeric biologically active form [3, 4]. It has been demonstrated that the glycosphingolipid, sulfated beta-galactosylceramide, also denominated sulfatide, acts as a chaperone for insulin during its folding prior to resolution into insulin crystals [5] and that sulfatide preserves these insulin crystals [5]. Within beta cells, sulfatide is present within the Golgi and insulin granules, as well as at the membrane surface [6].

The purpose of this current study was to characterize the relationship between insulin and sulfatide in order to gain additional understanding as to the importance of this association with metabolism and, potentially, aberrations related to this process that might influence health (i.e., diabetes, insulin resistance). The main question to be answered in this study is whether sulfatide is built into the crystals or is managing its preservation effect just by being attached to the surface of the insulin crystal.

## 2. Materials and Methods

### 2.1. Purification of Insulin

The human insulin gene (Novo Nordisk, Bagsvaerd, Denmark) was cloned into the pAK721 vector for expression in* Saccharomyces cerevisiae*. The native C-peptide was replaced by a synthetic minipeptide AAK, connecting the A- and B-chains, and pure insulin was obtained as earlier described [7].

### 2.2. Cocrystallization of Insulin-Sulfatide Complexes

Insulin and sulfatide (Avanti Polar Lipids, Inc., Alabaster, AL, USA) were mixed in a 1 : 3 molar ratio, and crystals were grown using the hanging drop vapor diffusion method [7, 8]. Preliminary screens were performed using sparse-matrix crystallization to determine initial conditions [9]. Crystals were grown in conditions that contain zinc acetate or magnesium sulfate as in previous studies [5]. Conditions were optimized based on the crystals grown in 1.6 mol/L magnesium sulfate and 0.1 mol/L MES pH 6.5. Large crystals (up to 200 *μ*m in the longest dimension) were obtained in five days in 1.3 mol/L magnesium sulfate and 0.1 mol/L MES pH 7.5. The single crystal used for the data reported was grown in a hanging drop containing 2 *μ*L protein solution (10 mg/mL + 10.3 mmol/L sulfatide) and 3 *μ*L reservoir solution. All crystallization trials were performed at 22°C.

### 2.3. X-Ray Data Collection

Data was collected on beamline X6A at the National Synchrotron Light Source (Brookhaven National Laboratories, Upton, NY, USA). Images were collected using an ADSC 210 CCD detector and indexed and scaled using HKL2000 [10]. XPREP [11] was used to assist space group determination. The selected insulin/sulfatide crystal was cryoprotected using paraffin oil, cooled in a stream of gaseous nitrogen. The crystal was mounted at a distance of 120 mm from the detector and at an X-ray wavelength of 0.9322 Å. 300 frames were collected, 8 seconds per image, with 0.5° oscillation steps for each frame. The molecular replacement software PHASER-MR implemented in PHENIX was used for phasing. PHENIX.REFINE: 1.9_1692 was used for refinement. PDB code 2INS [12] was the initial model used for phasing. Difference electron density maps were created comparing electron density of the crystal versus electron density explained by the model.

### 2.4. Electron Microscopy

Islet tissue was obtained from 9-week-old male Lewis rats purchased from Taconic Biosciences, Inc. (Hudson, NY, USA). The islets were isolated using a collagenase method [13]. Isolated islets were incubated overnight at 4°C with the sulfatide-specific monoclonal antibody, Sulph I (gift from Pam Fredman), diluted as 1 : 1000 [14], and after washing in 1% PBS-BSA, the islets were incubated overnight at 4°C with 1 nm gold labeled goat anti-mouse IgG (BBI Solutions, Cardiff, UK) diluted as 1 : 300 in 1% PBS-BSA and absorbed with rat serum. The islets were postfixed after washing in 2% glutaraldehyde for 2 h and washed in distilled water, before silver enhancement using AURION R-GENT SE-EM (Aurion, Wageningen, NL). The islets were washed in distilled water before osmication in 1% OsO4 diluted in 0.1 M cacodylate buffer. After washing in 0.1 M cacodylate buffer, the specimens were dehydrated in alcohol and embedded in Epon Resin 812 before ultra-sections were examined in a Philips 208 electron microscope.

## 3. Results

**Figure 1:**
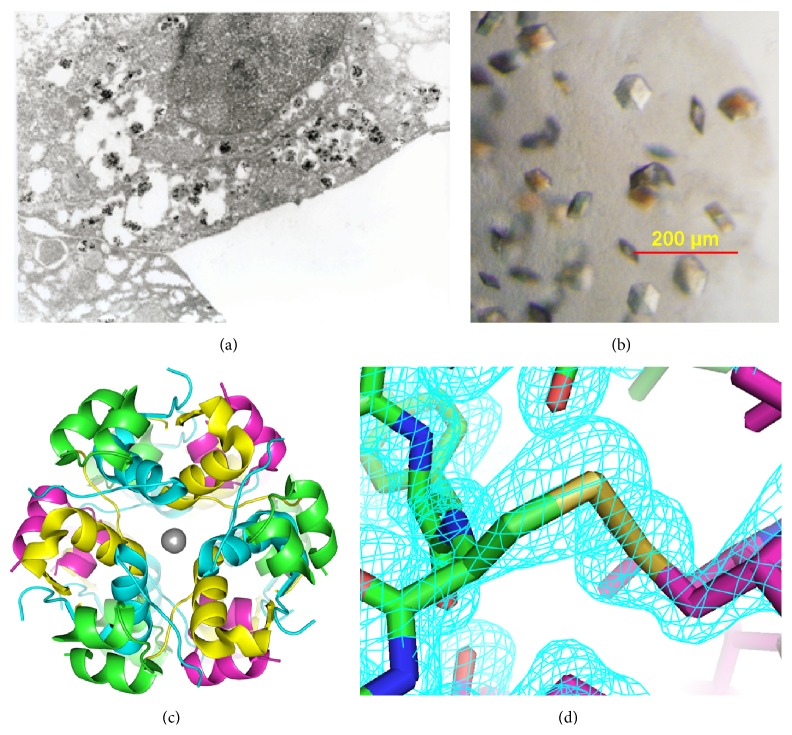
(a) Electron microscopy of a beta cell stained for sulfatide with colloidal gold granules as secondary indications. Close relationship to the insulin crystals is clearly seen. At the surface at the beta cells an insulin crystal close to exocytosis is clearly seen at one quartile from the right in the horizontal axis and half down in the vertical axis. (b) Upon crystallization, insulin formed hexamers of approximately 100 *μ*m diameter. ((c) and (d)) Structure of insulin crystallized in the presence of sulfatide shows classic features of zinc bound insulin hexamers. (c) The crystals structure of insulin (PDB code 4XC4). Zinc is depicted as gray spheres. (d) An electron density map surrounding the disulfide bond linking cysteine chain A 20 to cysteine chain B 19.

Staining of beta cells for sulfatide demonstrated a close relationship to the insulin crystals even at exocytosis (Figure 1(a)). As sulfatide has previously been noted to promote stability of insulin crystals within pancreatic beta cells [5], we investigated the molecular mechanisms supporting an integral role for sulfatide in insulin crystal formation and preservation. We cocrystallized human insulin with sulfatide in the presence of Zn (Figure 1(b)) and observed that crystals containing insulin bound to sulfatide appeared in a rhombohedral lattice, the same space group that has been reported for insulin molecules found in pancreatic tissue [15, 16]. The crystals were completely translucent to visible light and did not display precipitation either on the surface or within. X-ray diffraction data was collected to 1.6 Å resolution and reduced to a primitive rhombohedral lattice, H3 (Table 1). The unit cell parameters were *a* = *b* = 81.61 Å, *c* = 33.729 Å, with *α* = *β* = 90.0°, *γ* = 120.0°. Matthew's coefficient of 1.88 Å^3^ Da^−1^ is consistent with the presence of one insulin molecule per asymmetric unit [17].

The structure was solved by molecular replacement [12] and refined yielding statistics shown in Table 1, deposited in the Protein Data Bank as code 4XC4. Difference electron density maps revealed ordered water molecules and Zn in positions consistent with other hexameric insulin structures (Figures 1(c) and 1(d)) [5, 16]. Ordered electron density consistent with sulfatide was not observed. These data suggest that sulfatide does not stabilize insulin crystals through a mechanism involving stable contacts with insulin residues.

## 4. Discussion

In this study we have shown that sulfatide is closely related to and covers the insulin crystals* in vivo*. Furthermore, sulfatide is known to preserve insulin crystals in spite of our present findings that sulfatide is not integrated inside the matrix.

Beta cells must store large quantities of insulin within the secretory granules in preparation for normal blood glucose regulation. However, insulin has a tendency to fibrillate which renders it biologically inactive. For having enough storage, nature has solved this problem by crystallization of insulin that cannot fibrillate. Insulin is present in beta cells as hexamers that build into crystals that must be stable for weeks but simultaneously capable of being secreted immediately on demand of the blood glucose values. Thus, the insulin crystals should be able to break down very quickly, dissociating into monomers capable of binding the insulin receptor.

It has been previously demonstrated that at low pH (5.5) with high concentration of Zn^++^ insulin crystals are well preserved in presence of sulfatide [5]. At exocytosis of the insulin granules and thereby insulin secretion, the pH increases to 7.4 and the Zn^++^ concentration is lowered, potentiating monomerization. We hypothesized that sulfatide may act as a chaperone for insulin and preserve its crystals within the beta cell granule due to integration of the glycolipid into the insulin crystal. The present study suggests that sulfatide is coating the outside of the insulin crystals (as opposed to binding within the insulin crystal lattice), which is consistent with data showing close associations* in vivo*. The blood concentration of sulfatide has been noted to be lower in patients with type 2 diabetes [18], so sulfatide might offer a level of protection or improvement in insulin physiology. Further, sulfatide has been noted to be present at reduced quantities within the secretory granules of stressed beta cells [19]. Autoantibodies against sulfatide have been documented in patients with type 1 diabetes [20]. As being present at the surface of the insulin crystals only, variations in the amount of sulfatide might be critical for the beta cells. Thus, interactions between sulfatide and insulin crystals may have implications on disease pathogenesis in both type 1 and type 2 diabetes, which must be subject to further investigation.

## Figures and Tables

**Table 1:** X-ray diffraction data for insulin crystalized in the presence of sulfatide.

PDB 4XC4	
Resolution range (Å)	30–1.5 (1.553–1.499)
Space group	R3:H
Unit cell	*a* = 81.61, *b* = 81.61, and *c* = 33.73; *α* = 90, *β* = 90, and *γ* = 120
Total reflections (unique)	11,654 (1,124)
Completeness (%)	86.62 (82.71)
Mean *I*/sigma(*I*)	18.52 (2.60)
Wilson *B*-factor	20.27
R-work	0.2180 (0.3020)
R-free	0.2540 (0.3595)
Number of nonhydrogen atoms	870
Macromolecules	865
Residues	101
RMS (bonds)	0.008
RMS (angles)	1.06
Ramachandran favored (%)	95
Ramachandran allowed (%)	4.01
Ramachandran outliers (%)	0.99
*B*-factor	29.00
Macromolecules	29.00
Ligands	33.50
